# Clear phylogeographical structures shed light on the origin and dispersal of the aquatic boreal plant *Hippuris vulgaris*

**DOI:** 10.3389/fpls.2022.1046600

**Published:** 2022-11-28

**Authors:** Yinjiao Yu, Qixiang Lu, Alexander G. Lapirov, Joanna Freeland, Xinwei Xu

**Affiliations:** ^1^ National Field Station of Freshwater Ecosystem of Liangzi Lake, College of Life Sciences, Wuhan University, Wuhan, China; ^2^ Zhejiang Province Key Laboratory of Plant Secondary Metabolism and Regulation, College of Life Sciences and Medicine, Zhejiang Sci-Tech University, Hangzhou, China; ^3^ Papanin Institute for Biology of Inland Waters, Russian Academy of Sciences, Yaroslavl, Russia; ^4^ Department of Biology, Trent University, Peterborough, ON, Canada

**Keywords:** aquatic plants, arctic flora, *Hippuris vulgaris*, microsatellites, phylogeography, chloroplast DNA

## Abstract

Aquatic plants are an important ecological group in the arctic flora; however, their evolutionary histories remain largely unknown. In order to deepen our understanding of the evolution of these plants, we explored the phylogeographical structure of an aquatic boreal plant *Hippuris vulgaris* in a broad geographical sampling from Eurasia and North America using the chloroplast intergenic spacer *psb*A*-trn*H and seven nuclear microsatellite loci. Two closely-related species *H. lanceolata* and *H. tetraphylla* were also included because of their taxonomic controversy. Both chloroplast DNA sequences and nuclear microsatellite data revealed three genetic lineages with distinct distribution ranges. Incongruence between nuclear and chloroplast DNA lineages occurred in 14 samples from Russian Far East and Europe caused by inter-lineage hybridization. No private haplotypes or independent genetic clusters were evident in *H. lanceolata* or *H. tetraphylla*, suggesting that these two species should be considered conspecific ecotypes of *H. vulgaris*. Analysis using Approximate Bayesian Computation-Random Forest approach suggests that *Hippuris vulgaris* originated in China, followed by dispersal into Russia plus Northeast China, then successively westwards into Europe and North America, and finally into the Russian Far East from both North America and Russia plus Northeast China. This study is the first to elucidate the historical dispersal processes of a circumarctic aquatic plant across the entirety of its range.

## Introduction

The diversity and distributions of Arctic flora have been strongly influenced by millennia of expansive climate changes that led to repeated range contractions, evolution in distinct refugia, and large-scale dispersal (Abbott and Brochmann, 2003). The present distributions of the Arctic flora, which for many species are extensive, have therefore been influenced by their evolutionary histories (Brochmann and Brysting, 2008), the locations of glacial refugia (Westergaard et al., 2011), and dispersal patterns (Eidesen et al., 2013). Floristic analyses have indicated multiple origins of numerous arctic taxa, including *in situ* evolution of indigenous elements in the Arctic, range expansions from adjacent saline coastal and forest habitats, glacial dispersal across Beringian land bridges, and migration from high mountain ranges of the Northern Hemisphere (Hulteín, 1937; Tolmachev, 1960; Hedberg, 1992; Murray, 1995; Tkach et al., 2014; Maguilla et al., 2018). Thus, recent phylogeographical studies on multiple arctic species have revealed diversified patterns of their origins and formations (Alsos et al., 2005; Schönswetter et al., 2006; Ikeda et al., 2012; Winkler et al., 2012; Eidesen et al., 2013; Ikeda et al., 2014; Allen et al., 2015; Wang et al., 2016). However, these studies have mostly focused on terrestrial herbs, with less attention paid to aquatic plants. Aquatic plants comprise a significant group within the arctic flora (Hoffmann et al., 2010). Compared to terrestrial herbs, aquatic plants live in distinct habitats with special environmental conditions and thus are expected to have different evolutionary histories than terrestrial plants (Cook, 1990). Therefore, additional investigations into aquatic plants will deepen our overall understanding of the evolutionary histories of Arctic flora.

In this study, we focused on *Hippuris vulgaris* (Plantaginaceae), an aquatic plant of circumboreal distribution with its range extending into high mountains in Eurasia and North America (Elven et al., 2019). This species is a rhizomatous perennial that often grows in shallow waters of lakes, ponds and rivers. Three previous phylogeographical studies on *H. vulgaris* were conducted at different geographical scales: the Qinghai-Tibetan Plateau (Chen et al., 2013), China (Lu et al., 2016), and China plus a few sites in Europe and Japan (Dai et al., 2021). All three studies revealed high genetic diversity in the Qinghai-Tibetan Plateau, and Dai et al. (2021) inferred the Qinghai-Tibetan Plateau as the likely area of origin for *H. vulgaris*. However, because earlier studies did not include samples from Russia and North America, the question of how *H. vulgaris* attained a circumarctic distribution has not yet been resolved. Range-wide studies of broadly distributed species are relatively uncommon, and we thus have an incomplete understanding of how historical dispersal patterns have facilitated the success of broadly distributed taxa such as aquatic plants with circumarctic distributions.

In this study we substantially increased the range of sampled *H. vulgaris* in order to test three competing hypotheses: *H. vulgaris* originated in China and from there spread either eastwards, westwards, or both eastwards and westwards, until achieving its current circumarctic distribution. Our phylogeographical analysis of *H. vulgaris* is based on samples from Eurasia and North America that were genotyped on the basis of one chloroplast DNA fragment and seven nuclear microsatellite loci. Samples of two other putative *Hippuris* species, *H. lanceolata* and *H. tetraphylla*, were also included because of their taxonomic controversy. We identify these two species based on leaf morphology, although the plasticity of leaf forms suggests that these do not reliably identify taxa (McCully and Dale, 1961); however, this conclusion has been disputed by others who maintain that *H. lanceolata* and *H. tetraphylla* are distinct from *H. vulgaris* (Elven et al., 2019). It has also been suggested that *Hippuris lanceolata* is a hybrid between *H. vulgaris* and *H. tetraphylla*, based on its intermediate features (Tzvelev, 1980). Our aims were therefore to (1) reveal the phylogeographical structure of *H. vulgaris* and deduce its original area and dispersal routes, and (2) explore the genetic relationships among *H. vulgaris*, *H. lanceolata* and *H. tetraphylla* in order to add clarity to the taxonomy of *Hippuris*.

## Materials and methods

### Plant materials and DNA extraction

A total of 200 samples -157 of *H. vulgaris*, 33 of *H. lanceolata* and 10 of *H. tetraphylla* - were collected from 200 sites in Eurasia and North America. Some were collected in the field from 2017 to 2019, whereas others were from earlier collections that had been preserved on herbarium sheets (**Supplementary Table 1**). Samples were assigned to species based on their morphological characteristics (**Supplementary Figure 1**). Total genomic DNA was extracted from silica-dried leaves and herbarium specimens with the DNA Secure Plant Kit (Tiangen Biotech, Beijing, China).

### Amplification, sequencing and genotyping

We generated sequence data from the chloroplast DNA fragment *trn*H-*psb*A using primers trnH^GUG^ (Tate and Simpson, 2003) and psbA (Sang et al., 1997) for all 200 samples (after Shaw et al., 2005). For 105 samples we also generated data from seven nuclear microsatellite loci (Hpv11, Hpv22, Hpv27, Hpv30, Hpv37, Hpv67 and Hpv75; Lu et al., 2016) (**Supplementary Table 1**). PCR amplifications, sequencing and genotyping were performed following the methods outlined in Lu et al. (2016).

### Analyses of cpDNA sequence data

All cpDNA sequences were aligned by MAFFT v7.3.1 (Katoh and Toh, 2008). Sequences were collapsed into haplotypes using DnaSP v5.0 (Librado and Rozas, 2009). Sequences of haplotypes were deposited in GenBank (see **Supplementary Table 1** for accession numbers). The relationships among haplotypes were analyzed by constructing a network based on a median-joining algorithm (Bandelt et al., 1999) in NETWORK v4.0 (http://www.fluxus-engineering.com). The phylogeny of haplotypes was reconstructed using maximum likelihood (ML) analysis implemented in GARLI v0.951 (Zwickl, 2006). The substitution model was identified using the Akaike information criterion in modelgenerator v.851 (Frech et al., 1997). One thousand bootstrap replicates were run in GARLI to estimate the bootstrap support. Bayesian inference (BI) implemented in MrBayes v3.1.2 (Ronquist et al., 2012) was also used for phylogenetic reconstruction. Two independent Markov Chain Monte Carlo (MCMC) analysis runs, with each including one cold and three hot chains, were conducted simultaneously beginning with a random tree. One million generations were run with sampling at every 1000 generations. Chain convergence was checked using Tracer v1.5 (Rambaut and Drummond, 2007), and the first 25% of samples were discarded as burn-in. The congeneric species *H. montana*, plus three species in the family Plantaginaceae - *Callitriche lenisulca*, *Streptocarpus teitensis* and *Plantago ovata* - were included as outgroups in the phylogenetic analyses.

### Analyses of nuclear microsatellite loci data

Microsatellite loci data were obtained from a subset of 105 individuals, due to the difficulty of PCR amplification from herbarium materials. Genetic clusters among 105 individuals (26 *H. lanceolata*, 7 *H. tetraphylla*, and 72 *H. vulgaris*) were inferred by the software STRUCTURE v2.3.4 (Pritchard et al., 2000). The number of clusters (K) was set from 1 to 10. Under the admixture model, ten independent runs were performed for each K value with 300,000 MCMC iterations and a burn-in period of 100,000 iterations. The optimal number of clusters was determined based on the value of ΔK (Evanno et al., 2005). Prior to Structure analysis, we used SHEsis software (Shi and He, 2005) to detect linkage disequilibrium among these seven microsatellite loci to see if they met the prerequisites for Structure analysis. Principal coordinate analysis (PCoA), implemented in GenALEx v6.5 (Peakall and Smouse, 2012), was also used to compare the genetic similarities among individuals.

The demographic history of *H. vulgaris* was inferred based on nuclear microsatellite loci data using an Approximate Bayesian Computation-Random Forest (ABC-RF) approach implemented in DIYABC Random Forest v1.0 (Collin et al., 2021). ABC-RF outperforms other ABC methods by offering a significant improvement in robustness to the choice of summary statistics (Raynal et al., 2019). Samples were divided into five groups for ABC-RF simulation on the basis of their genetic clusters corresponding to geographical distributions: North America, Europe, Russia plus Northeast China, Russian Far East, and China (see Results for details). We modelled 20 possible evolutionary scenarios (**Supplementary Figure 2**) with China (scenarios 1-8), North America (scenarios 9-14) and Europe (scenarios 15-20) each considered a potential area of origin. Neither Russia plus Northeast China nor Russian Far East was considered a potential ancestral site because the origin of ancient arctic plant in either of these areas is considered unrealistic based on previous studies (Liu et al., 2014; Tkach et al., 2014; Wang et al., 2016). We performed three ABC-RF analyses. For the first analysis, these 20 scenarios were analysed individually. For the second analysis, scenarios were combined into three groups based on different original areas, with group 1 consisting of scenarios considering China as the original area (scenarios 1-8), group 2 including scenarios considering North America as the original area (scenarios 9-14) and group 3 containing scenarios considering Europe as the original area (scenarios 15-20). To improve the robustness of the first analysis (see Results), we performed the third analysis only for scenarios 1-8 individually because group 1 was selected as the best fit in the second analysis (see Results). In all three analyses, 100,000 training datasets were generated for each scenario and the number of trees in the random forest was set to 1000. The settings for all prior parameters are listed in **Supplementary Table 2**. The posterior probability, as well as the global and local error rates were used to assess the choice of scenario and the quality of prediction (Collin et al., 2021). Five independent runs were performed for each analysis. Population regeneration of *H. vulgaris* can be achieved through sexual reproduction and vegetative propagation. Because existing coalescent simulators for microsatellite loci data cannot simulate populations with different recombination rates simultaneously (e.g. Cornuet et al., 2014; Zhu et al., 2020), the divergence time among groups was not estimated here.

## Results

### Chloroplast DNA phylogeography

**Figure 1:**
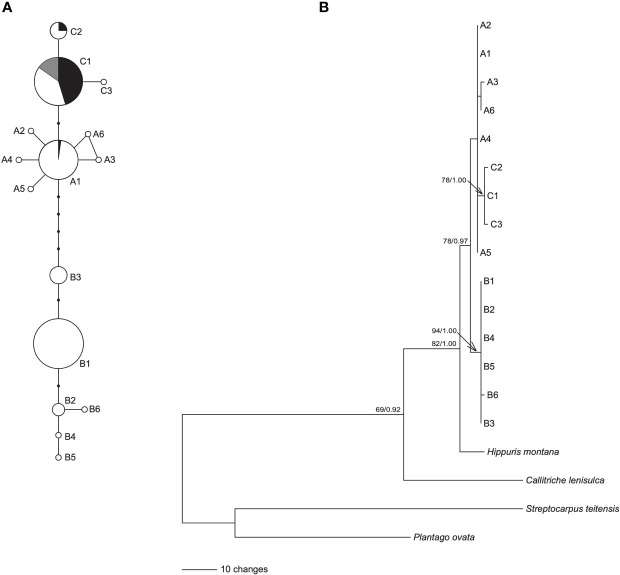
Network and phylogenetic tree of 15 cpDNA haplotypes of *Hippuris vulgaris*. **(A)** Network of genealogical relationships among the 15 cpDNA haplotypes. The black dots represent missing haplotypes. Three haplotypes, A1, C1 and C2, were also from (*H. lanceolata* and *H. tetraphylla*, and their proportions were indicated by black and grey, respectively. **(B)** Phylogenetic tree of 15 cpDNA haplotypes inferred using GARLI. Numbers at nodes are the ML bootstrap values (BS) and Bayesian posterior probabilities (PP) in phylogeny reconstruction. The nodes without numbers indicate BS < 65 and PP < 0.90.

**Figure 2:**
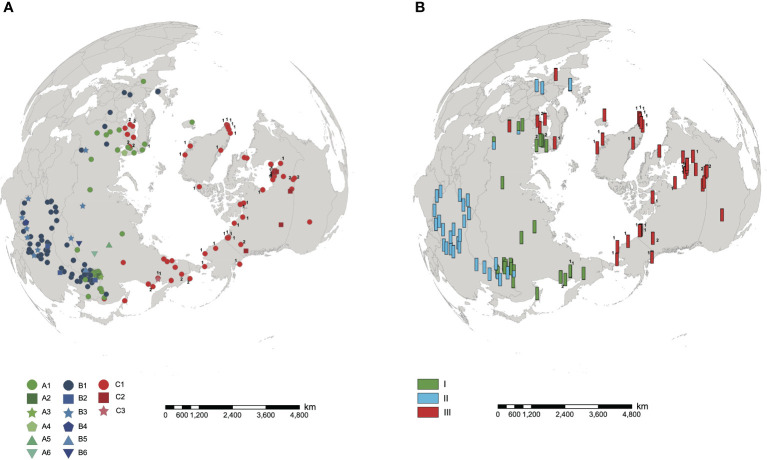
Distribution of cpDNA haplotypes and nuclear microsatellite clusters in *Hippuris vulgaris*. Samples of *H. lanceolata* and *H. tetraphylla* were marked with the number 1 and 2, respectively. **(A)** Distribution of 15 cpDNA haplotypes. **(B)** Distribution of three nuclear microsatellite clusters identified using STRUCTURE.

The length of aligned sequences from 200 individuals was 443bp. The sequences were collapsed into 15 haplotypes: A1-A6, B1-B6 and C1-C3. These haplotypes were divided into lineage AC and lineage B, based on the haplotype network (**Figure 1A**) and phylogenetic tree (**Figure 1B**). Lineage AC is further subdivided into lineage A and lineage C because the phylogeny identifies lineage C as monophyletic (**Figure 1B**), and on the haplotype network lineages A and C form two discrete clusters (**Figure 1A**). Haplotype C1 was identified in each of the 10 samples of *H. tetraphylla*, and haplotypes A1, C1, and C2 were identified across the 33 samples of *H. lanceolata*; all of these haplotypes were also identified in *H. vulgaris* (**Figure 1A**, **Supplementary Table 1**). Lineage A is distributed in the northern range of Eurasia: haplotype A1 is widespread and present at sites in Europe, Russia and China, whereas each of the other five A haplotypes is restricted to a single site in Russia or China (**Figure 2A**). Lineage B occurred in the southern range of Eurasia. The most common haplotype, B1, was present at sites in Europe, Russia and China, whereas haplotype B2 occurred at 3 sites in China, and haplotype B3 was found at 9 sites in China, Russia and Mongolia. Haplotypes B4, B5 and B6 each occurred at one site in either China or Russia (**Figure 2A**). Lineage C comprised three haplotypes, with haplotype C1 widespread in North America, Northern Europe, and Russian Far East. Haplotype C2 was found at 8 sites in Canada, and haplotype C3 was restricted to a single site in the Russian Far East (**Figure 2A**).

The ML and BI trees showed the same topology. The 15 haplotypes formed a monophyletic lineage with robust support, which divided into two clades: clade B consisted of haplotypes B1-B6 with robust support, and clade A+C included haplotypes A1-A6 and C1-C3 (**Figure 1B**). Furthermore, haplotypes C1-C3 formed a monophyletic lineage with high support nested in clade A+C (**Figure 1B**).

### Nuclear DNA phylogeography

**Figure 3:**
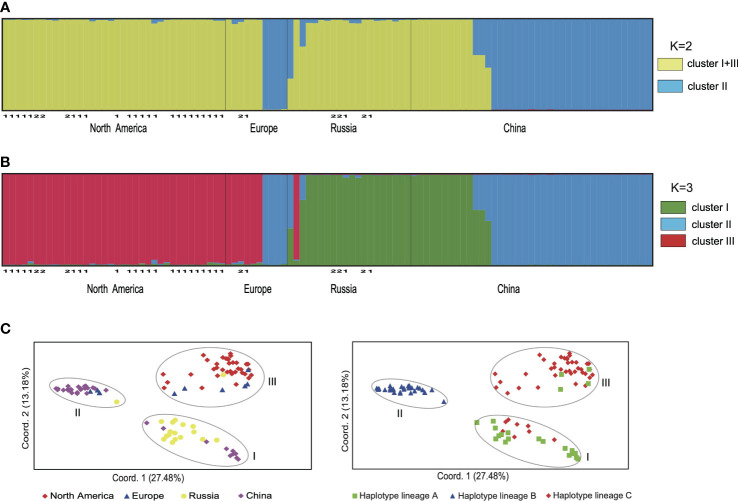
The genetic clusters of *Hippuris vulgaris* based on nuclear microsatellite data. **(A)** The bar plot depicts the STRUCTURE admixture coefficients for individuals when K = 2. Samples of *H. lanceolata* and *H. tetraphylla* were marked with the number 1 and 2, respectively. **(B)** The bar plot depicts the STRUCTURE admixture coefficients for individuals when K = 3. Samples of *H. lanceolata* and *H. tetraphylla* were marked with the number 1 and 2, respectively. **(C)** Principal coordinate analysis performed from pairwise genetic distances among individuals. Individuals were classified by their geographical origins (left) or chloroplast DNA haplotype lineages (right).

The results of SHEsis analysis indicated the seven microsatellite loci were unliked based on the very low values of r^2^ (**Supplementary Table 3**). The STRUCTURE analyses of microsatellite loci data from 105 individuals suggested that K=2 was the most likely number of genetic clusters, with K=3 as the second most likely based on the calculation of ΔK (**Supplementary Figure 3**). When K=2, the first cluster comprised samples from North America, Northeast China, Europe and Russia, which included individuals with cpDNA haplotypes from lineages A and C. The second cluster included samples from China, Europe and Russia (**Figure 3A**, **Supplementary Figure 4**), all of which had cpDNA haplotypes from lineage B (**Supplementary Table 1**). Five samples from China and Russia were admixtures of these two genetic clusters with a probability ranging from 0.302 to 0.698 (**Figure 3A**). When K=3, we defined these three clusters as I, II, and III. Clusters I + III combined corresponded to the first cluster when K=2, and cluster II corresponded to the second cluster when K=2 (**Figure 3B**). Cluster I included samples from Northeast China and Russia mainly corresponding to haplotype lineage A and also included 10 samples from Russian Far East with haplotype C1 or C3. Cluster III included samples from North America and Europe mainly corresponding to haplotype lineage C and also included four samples from Europe and Russia with haplotype A2 (**Figures 2**, **3**; **Supplementary Table 1**). The genotypes of neither *H. lanceolata* nor *H. tetraphylla* were assigned into independent genetic clusters (**Figures 3A, B**; **Supplementary Table 1**). Similarly, the PCoA analysis revealed three genetic groups (**Figure 3C**), which corresponded to clusters I, II, and III revealed by STRUCTURE.

**Figure 4:**
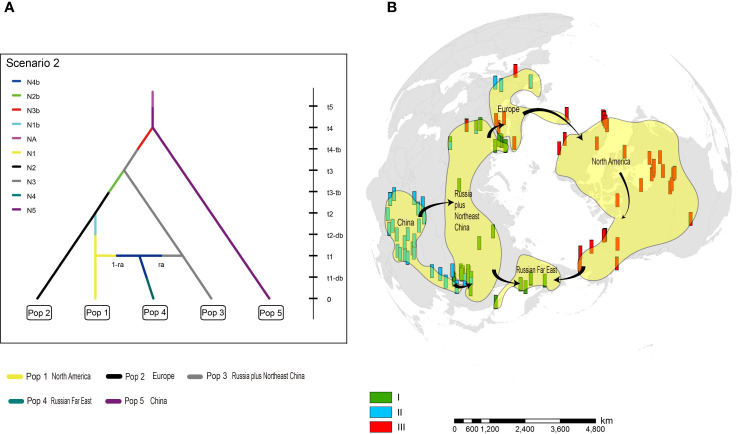
The origin and dispersal of *Hippuris vulgaris*. **(A)** The best fit model (Scenario 2) of divergence within *H. vulgaris* analysed by ABC-RF. **(B)** Most likely scenario of dispersal routes for *H. vulgaris* to attain a circumarctic distribution.

Among 20 alternative scenarios of the demographic history of *H. vulgaris*, ABC-RF modelling based on microsatellite loci data indicated scenario 2 was the best supported model, based on the highest mean classification vote of 263.8 and mean posterior probability of 0.476, with global and local error rates of 0.746 and 0.524, respectively (**Table 1**). When the scenarios were analysed in three groups, group 1, which supports China as the original area, was selected as the best fit, with mean posterior probability of 0.698 accompanied by global and local errors of 0.342 and 0.302, respectively (**Table 1**). To improve prediction quality, we ran the third analysis for scenarios 1-8 in group 1. Scenario 2 was selected as the best fit again, with mean posterior probability of 0.551 accompanied by global and local errors of 0.653 and 0.449, respectively (**Table 1**). For scenario 2, China was identified as the ancestral area, and the populations spread from China to Russia plus Northeast China. European populations descended from Russia and dispersed to North America. Populations from Russian Far East were derived from plants that migrated from both North America and Russia plus Northeast China (**Figure 4**).

**Table 1:** Results of scenario choice in three DIYABC-RF analyses.

Analysis	Global	Local	Votes scenario:										Posterior
	error	error	1	2	3	4	5	6	7	8	9	10	probability
	rate	rate	11	12	13	14	15	16	17	18	19	20	
20 scenarios	0.746 (0)	0.524 (0.009)	28.2 (6.06)	263.8 (18.78)	31.8 (4.55)	146.2 (8.87)	51 (5.61)	20.6 (3.58)	39.2 (2.86)	50.4 (3.21)	8.6 (2.88)	7.6 (3.58)	0.476 [scen. 2] (0.009)
			4.4 (3.36)	11.2 (4.15)	7.4 (3.51)	87.4 (7.09)	90.6 (4.10)	9.4 (2.79)	94.2 (9.23)	7.6 (0.89)	11.4 (4.34)	29 (4.30)	
Three groups	0.342 (0)	0.302 (0.020)	596.4 (14.10)	158.8 (10.69)	244.8 (13.95)								0.698 [group 1] (0.020)
Eight scenarios in group 1	0.653 (0)	0.449 (0.021)	45 (3.08)	413.8 (15.02)	39.2 (3.11)	283.6 (13.76)	78 (8.57)	23.8 (2.78)	53.2 (6.98)	63.4 (5.37)			0.551 [scen. 2] (0.021)

The values are mean values over the five replicates for each of the analyses with standard deviation over the five replicates provided in parentheses for each metric.

## Discussion

### Phylogeographical structure

Many arctic plants have distinct lineages and obvious phylogeographical structures, such as *Oxyria digyna* (Wang et al., 2016), *Sibbaldia procumbens* (Allen et al., 2015), *Saxifraga oppositifolia* (Winkler et al., 2012), and *Dryas octopetala* (Skrede et al., 2006). *Hippuris vulgaris* similarly shows phylogeographic structuring, although with more widespread haplotypes compared with these terrestrial herbs: each *H. vulgaris* lineage included a haplotype (A1, B1 and C1, respectively) that was broadly distributed throughout Eurasia and/or North America (**Figure 2A**). Because of the limited life span of wetlands on geological and evolutionary time scales, dispersal (chiefly mediated by birds) rather than vicariance has often been used to explain the broad distribution of aquatic plants (Barrett et al., 1993; Santamaría, 2002). High clonality is another explanation for the broad distribution of aquatic plants because it reduces the risk of genotype mortality and the genetic differentiation among populations (Barrett et al., 1993). *Hippuris* is common in the diet of waterbirds (Dessborn et al., 2011). Therefore, the same haplotype of *H. vulgaris* could disperse to multiple sites mediated by birds and persist there for a long time by high levels of asexual reproduction.

Chloroplast DNA sequences and nuclear microsatellite data each identified three genetic lineages of *H. vulgaris* distributed across different regions (**Figures 2**, **3**). However, incongruence between these two molecular markers occurred in 14 samples from Russian Far East and Europe (**Figures 2**, **3**; **Supplementary Table 1**). Both incomplete lineage sorting and hybridization/introgression can cause phylogenetic incongruence between nuclear and plastid DNA (Wendel and Doyle, 1998). In *H. vulgaris*, the two atypical combinations of nuclear and plastid DNA (cluster I/lineage C and cluster III/lineage A, **Figure 3C**) most likely arose following inter-lineage hybridization and subsequent backcrossing which led to cytonuclear discordance, a phenomenon that has been reported in many plant lineages (Fehrer et al., 2007; Acosta and Premoli, 2010; Folk et al., 2017; Liu et al., 2020). Additionally, some individuals in the contact area of clusters I and II showed evidence of mixed ancestry, which is also suggestive of inter-lineage hybridization (**Figures 2B**, **3B**). However, in this case hybridization was likely more recent because for these hybrids the values of the admixture coefficients are largely consistent with F1 hybrids (**Figure 3B**). Further detailed studies are needed to explore the hybridization patterns among lineages.

Taxonomic controversy has surrounded *H. vulgaris*, *H. lanceolata* and *H. tetraphylla*. McCully and Dale (1961) proposed that their morphological differences reflected phenotypic plasticity and thus they should be treated as a single species. Tzvelev (1980) speculated that *H. lanceolata* is a hybrid of *H. vulgaris* and *H. tetraphylla* due to its intermediate features. However, Elven et al. (2019) concluded that clear morphological limits and discontinuities in leaf number and shape identify them as three different species. Our study revealed no private haplotypes or independent genetic clusters for *H. lanceolata* and *H. tetraphylla* (**Figures 1**, **2**, **3**; **Supplementary Table 1**) although samples of the two putative species with typical species morphological characters (**Supplementary Figure 1**) were included; these results support the conclusion of McCully and Dale (1961) that *H. lanceolata* and *H. tetraphylla* should be merged into *H. vulgaris*. However, McCully and Dale (1961) only used North American samples, and our data based on widespread sampling revealed three genetic lineages of *H. vulgaris* with different geographical distributions (**Figure 2**). Whether the three lineages merit taxonomic recognitions need to be determined in future studies that provide data on morphological variation and from more genetic markers.

### Biogeographical history of *Hippuris vulgaris*

ABC-RF analysis of nuclear microsatellite data identified China as the ancestral area for *H. vulgaris*. The more specific region is likely the Qinghai-Tibet Plateau (QTP) because populations in the QTP have higher genetic diversity than those in Northwest China and Northeast China (Lu et al., 2016). The QTP origin is characteristic of many arctic plants (e.g. Guest and Allen, 2014; Li et al., 2014; Allen et al., 2015; Wang et al., 2016). After originating in China and then dispersing northwards and westwards into Europe, further diversification of *H. vulgaris* likely occurred when Clusters I and III diverged in Europe, far from the QTP (**Figure 2B**). Europe is also identified as a diversification center for some additional species of arctic plants (e.g. Schönswetter et al., 2003; Winkler et al., 2012). Our model further suggests that Cluster III spread into North America, and then moved southwards and westwards across North America. It is noteworthy that although scenario 2 was identified as the best model for *H. vulgaris*, the large value of the local (posterior) error rate (0.524 or 0.449) indicates that future investigations based on a larger number of loci are necessary to give a more reliable answer (Pudlo et al., 2016).

The speciation and diversification of other arctic plant species that originated in the QTP (as discussed above) occurred across multiple geological ages, and were likely triggered and facilitated by various QTP uplift events between the early Miocene and the Quaternary (Wen et al., 2014). Although the divergence time of *H. vulgaris* lineages could not be estimated here, fossil records, which include one of Plio-Pleistocene age from North Greenland (Bennike and Böcher, 1990) and another of middle and upper Pleistocene age from central England (Kelly, 1968), suggest that lineages diverged in the Pliocene or earlier. A previous study (Lu et al., 2016) dated the divergence of lineages A and B to the early Pleistocene (ca. 1.36 Ma) based on an evolutionary rate of chloroplast intergenic regions calculated from sugarcane, maize and rice (Yamane et al., 2006). However, the dominance of asexual reproduction and clonal growth in populations of *H. vulgaris* mean that its molecular evolutionary rate is likely far lower than that of gramineous herbs. Therefore, it is necessary to conduct further studies to accurately estimate the divergence time of *H. vulgaris* lineages.

Dai et al. (2021) similarly concluded that QTP was the likely origin of *H. vulgaris*, although their sampling focused largely on plants from China, with relatively few samples from West Europe and none from North America, meaning that their inferences were based on only two cpDNA lineages. By sampling across a wide range in North America in addition to Europe and Asia, we were able to identify three cpDNA lineages compared to the two that Dai et al. (2021) uncovered, which in turn provided insight into the diversification and spread of *H. vulgaris*. Dai et al. (2021) concluded from two lineages that the most likely historical dispersal route was from Japan to North American, possibly across the Bering Land Bridge, and then across the Atlantic Ocean or North Atlantic Land Bridge to Europe. Conversely, our modelling based on three lineages suggests that *H. vulgaris* spread from China to Russia, then successively westwards to Europe and North America, and finally from both North America and Russia plus Northeast China to the Russian Far East. The different conclusions reached by Dai et al. (2021) and this study illustrate the importance of sampling from throughout the range when investigating extremely widespread taxa such as *H. vulgaris*.

Many aquatic boreal plants have extremely large geographical distributions, although relatively few have been studied throughout their range. In this study, we have demonstrated that comprehensive sampling across multiple continents is necessary before reconstructing the evolutionary histories of lineages; once reconstructed, these histories can provide substantial insights into historical patterns of dispersal and colonization. Future studies based on samples from additional species that follow circumarctic distributions could investigate the potential influences of additional factors such as life history and reproductive strategies on the historical diversification and colonization of populations.

## Data availability statement

The data presented in the study are deposited in the National Center for Biotechnology Information (GenBank) under accession numbers OK500317-OK500331, https://www.ncbi.nlm.nih.gov/genbank/.

## Author contributions

JF and XX designed the study. QL, AL, JF and XX performed the field work. YY and QL conducted experiments and analyzed the data. YY, AL, XX and JF wrote the manuscript. All authors contributed to the article and approved the submitted version.

## Funding

This study was supported by the China Scholarship Council (contract 201606275169), National Natural Science Foundation of China (31700321) and an NSERC discovery grant to JRF (RGPIN-2017-04371).

## Acknowledgments

We especially thank Michael Oldham, Bruce Bennett, Eric Snyder, Gabriel Lanthier, Paul Sokoloff, Polina Volkova, Lynn Gillespie, Jennifer Doubt, and Richard Fedorak for their help in collecting samples. We also thank B.A. Bennett Herbarium, Yukon (BABY); the Canadian Museum of Nature, Ottawa; and the Papanin Institute for Biology of Inland Waters Russian Academy of Sciences, Russia, for permission to remove tissue from herbarium specimens.

## Conflict of interest

The authors declare that the research was conducted in the absence of any commercial or financial relationships that could be construed as a potential conflict of interest.

## Publisher’s note

All claims expressed in this article are solely those of the authors and do not necessarily represent those of their affiliated organizations, or those of the publisher, the editors and the reviewers. Any product that may be evaluated in this article, or claim that may be made by its manufacturer, is not guaranteed or endorsed by the publisher.
