# Supplementary material for: Clear phylogeographical structures shed light on the origin and dispersal of the aquatic boreal plant Hippuris vulgaris
Source: Front Plant Sci. 2022 Nov 28;13:1046600. doi: 10.3389/fpls.2022.1046600 (PMC9742601; doi:10.3389/fpls.2022.1046600)
Supplement: Supplementary file 1 [file DataSheet_1.docx]

Supplementary Material


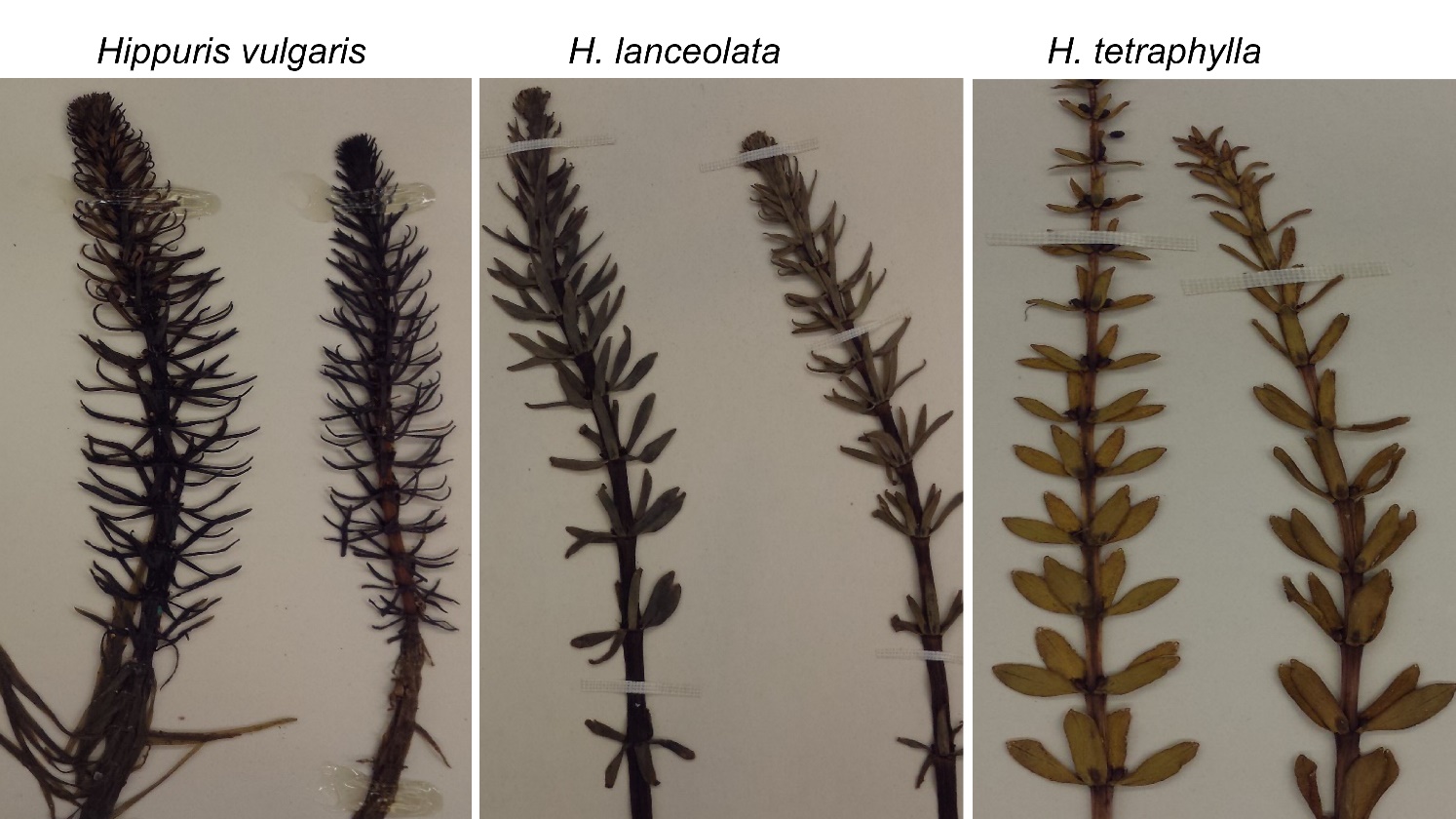


**Supplementary Figure 1.** Specimen images of *Hippuris vulgaris*, *H. lanceolata* and *H. tetraphylla* from the herbarium of Ministry of Natural Resources and Forestry, Ontario, Canada. The collectors and collecting number of these three specimens are M.J. Oldham & D.A. Sutherland 16758, M.J. Oldham, M.A. Young & J. Belliveau 40799, and M.J. OIdham & D.A. Sutherland 24890, respectively.


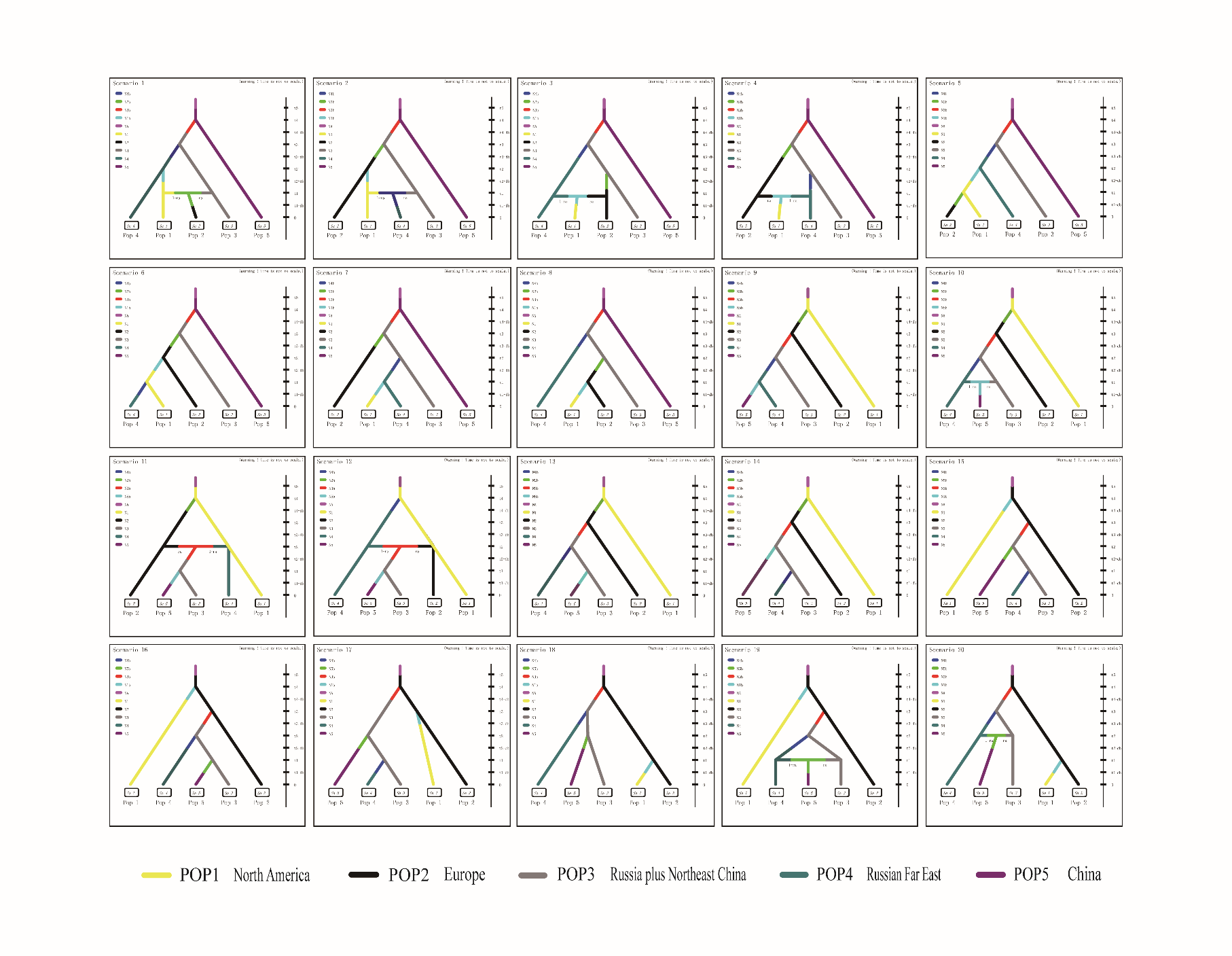


**Supplementary Figure 2.** Alternative demographic models (20 scenarios) of divergence within *Hippuris vulgaris* analysed by ABC-RF.


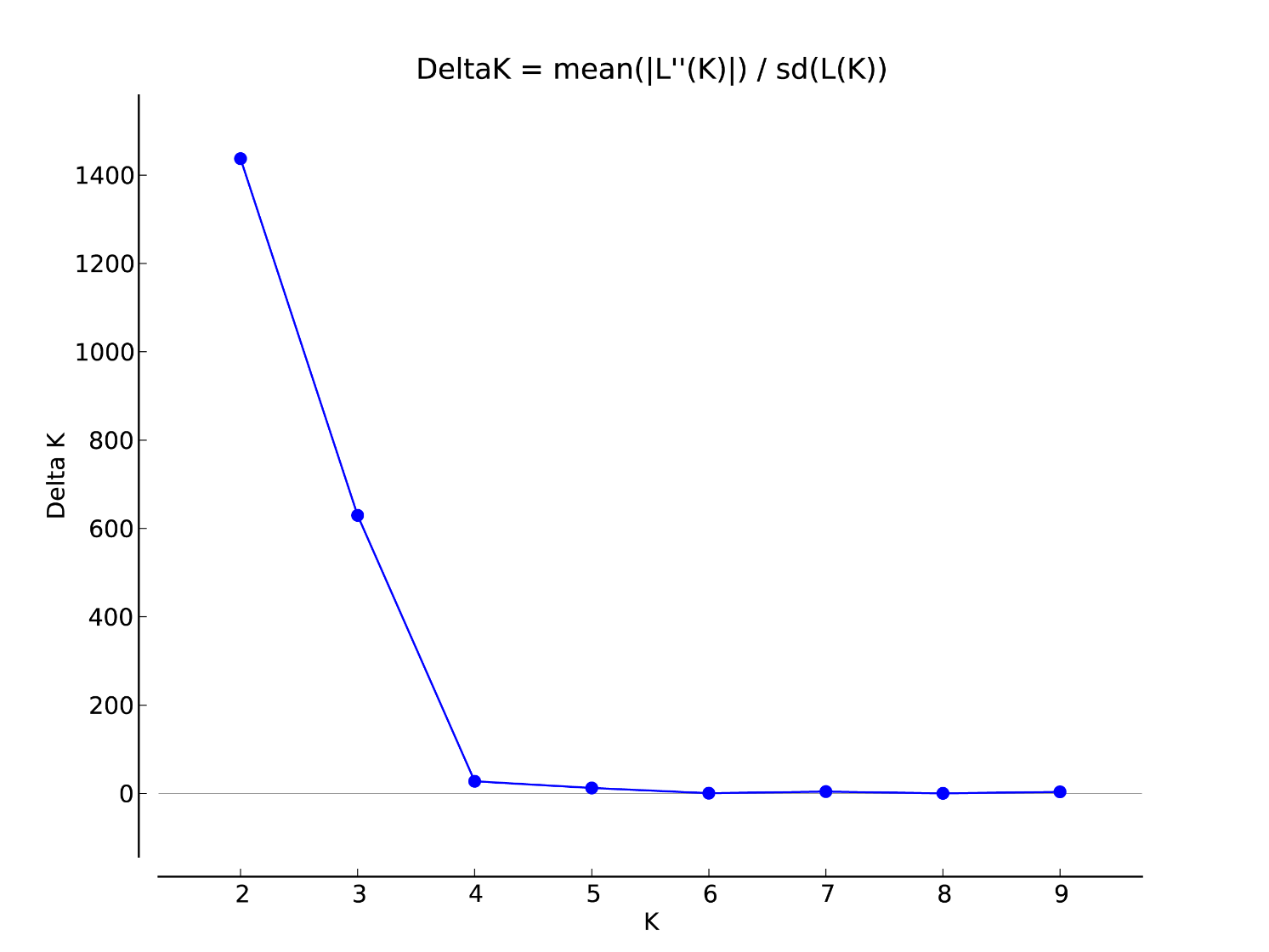


**Supplementary Figure 3**. Modelling of the number of genetic clusters in *Hippuris vulgaris* using STRUCTURE. ΔK values were calculated based on Evanno *et al*. (2005), charted against the number of modeled genepools (K)


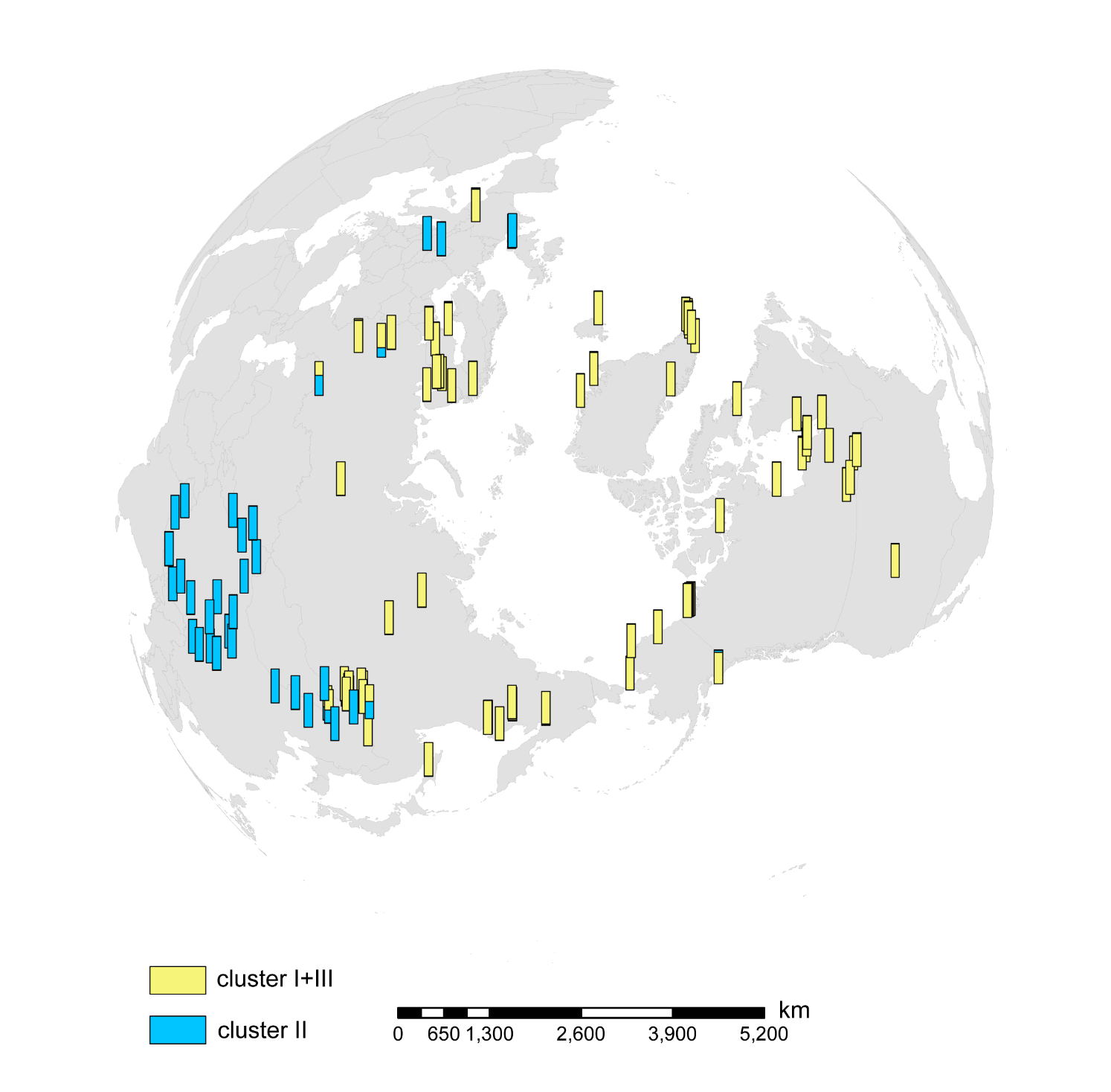


**Supplementary Figure 4**. Distribution of two nuclear microsatellite clusters in *Hippuris vulgaris*

**Supplementary Table 1.** The collection information and genetic assignment of *Hippuris* samples

| Species | Collection No. | Latitude | Longitude | Country/ region | cpDNA haplotype (Accession No.) | nSSR cluster |
| --- | --- | --- | --- | --- | --- | --- |
| *H. vulgaris* | Xu495 | 50.67 | 122.13 | China | A1 (OK500317) | I |
| *H. vulgaris* | Xu501 | 50.77 | 121.50 | China | A1 | I |
| *H. vulgaris* | Xu514 | 50.57 | 121.05 | China | A1 | I |
| *H. vulgaris* | Xu3977 | 48.47 | 129.67 | China | A1 | I |
| *H. vulgaris* | Xu4542 | 52.86 | 123.33 | China | A1 | I |
| *H. vulgaris* | Xu4551 | 52.94 | 122.57 | China | A1 | I |
| *H. vulgaris* | Xu4561 | 51.14 | 121.27 | China | A1 | I |
| *H. vulgaris* | Xu4578 | 51.01 | 120.05 | China | A1 | I |
| *H. vulgaris* | Xu6161 | 50.15 | 121.83 | China | A1 | I |
| *H. vulgaris* | Xu547 | 48.51 | 122.15 | China | A1 | / |
| *H. vulgaris* | Xu278 | 47.71 | 130.94 | China | A1 | / |
| *H. vulgaris* | Xu3802 | 42.05 | 127.75 | China | A1 | / |
| *H. vulgaris* | Xu3881 | 46.35 | 133.56 | China | A1 | / |
| *H. vulgaris* | Xu3976 | 48.19 | 129.35 | China | A1 | / |
| *H. vulgaris* | Xu4536 | 52.48 | 124.62 | China | A1 | / |
| *H. vulgaris* | Xu4552 | 52.21 | 122.14 | China | A1 | / |
| *H. vulgaris* | Xu4560 | 51.47 | 121.62 | China | A1 | / |
| *H. vulgaris* | Xu4582 | 50.83 | 119.91 | China | A1 | / |
| *H. vulgaris* | Xu4627 | 46.90 | 120.25 | China | A1 | / |
| *H. vulgaris* | Xu7151 | 50.73 | 124.33 | China | A1 | / |
| *H. vulgaris* | Xu7167 | 52.10 | 123.34 | China | A1 | / |
| *H. vulgaris* | Xu7184 | 50.77 | 121.50 | China | A1 | / |
| *H. vulgaris* | Xu7189 | 49.86 | 121.32 | China | A1 | / |
| *H. vulgaris* | Xu7228 | 46.51 | 121.38 | China | A1 | I+II |
| *H. vulgaris* | Xu4633 | 46.71 | 120.72 | China | A1 | I+II |
| *H. vulgaris* | Xu7157 | 51.84 | 124.54 | China | A2 (OK500318) | I |
| *H. vulgaris* | Xu4519 | 51.99 | 126.25 | China | A3 (OK500319) | I+II |
| *H. vulgaris* | Xia96 | 46.49 | 90.20 | China | B1 (OK500323) | II |
| *H. vulgaris* | Xu2345 | 29.28 | 100.08 | China | B1 | II |
| *H. vulgaris* | Xu2360 | 32.50 | 102.37 | China | B1 | II |
| *H. vulgaris* | Xu2386 | 33.26 | 103.76 | China | B1 | II |
| *H. vulgaris* | Xu2537 | 43.63 | 93.19 | China | B1 | II |
| *H. vulgaris* | Xu2583 | 43.33 | 115.68 | China | B1 | II |
| *H. vulgaris* | Xu4256 | 46.67 | 83.93 | China | B1 | II |
| *H. vulgaris* | Xu4315 | 43.16 | 81.50 | China | B1 | II |
| *H. vulgaris* | Xu4446 | 44.51 | 86.00 | China | B1 | II |
| *H. vulgaris* | Xu5007 | 37.61 | 101.32 | China | B1 | II |
| *H. vulgaris* | Xu5030 | 37.54 | 95.43 | China | B1 | II |
| *H. vulgaris* | Xu5058 | 31.73 | 94.49 | China | B1 | II |
| *H. vulgaris* | Xu5082 | 34.72 | 98.10 | China | B1 | II |
| *H. vulgaris* | Xu5135 | 30.81 | 81.56 | China | B1 | II |
| *H. vulgaris* | Xu5177 | 28.06 | 91.95 | China | B1 | II |
| *H. vulgaris* | Xu6604 | 37.38 | 102.96 | China | B1 | II |
| *H. vulgaris* | Xu6646 | 39.74 | 98.55 | China | B1 | II |
| *H. vulgaris* | Xu6762 | 40.88 | 112.78 | China | B1 | II |
| *H. vulgaris* | Xu6802 | 43.23 | 119.55 | China | B1 | II |
| *H. vulgaris* | Xu7023 | 45.20 | 124.45 | China | B1 | II |
| *H. vulgaris* | Xu7214 | 48.41 | 117.57 | China | B1 | II |
| *H. vulgaris* | Xia11 | 42.19 | 116.48 | China | B1 | / |
| *H. vulgaris* | Xia125 | 46.84 | 86.12 | China | B1 | / |
| *H. vulgaris* | Xu517 | 50.39 | 120.63 | China | B1 | / |
| *H. vulgaris* | Xu525 | 49.90 | 120.02 | China | B1 | / |
| *H. vulgaris* | Xu556 | 48.00 | 123.06 | China | B1 | / |
| *H. vulgaris* | Xu639 | 47.74 | 124.55 | China | B1 | / |
| *H. vulgaris* | Xu2329 | 27.50 | 99.82 | China | B1 | / |
| *H. vulgaris* | Xu2451 | 28.51 | 98.91 | China | B1 | / |
| *H. vulgaris* | Xu2456 | 30.02 | 97.04 | China | B1 | / |
| *H. vulgaris* | Xu2515 | 34.24 | 102.33 | China | B1 | / |
| *H. vulgaris* | Xu3902 | 48.07 | 133.40 | China | B1 | / |
| *H. vulgaris* | Xu4275 | 44.98 | 81.03 | China | B1 | / |
| *H. vulgaris* | Xu4285 | 44.56 | 81.35 | China | B1 | / |
| *H. vulgaris* | Xu4589 | 50.65 | 119.38 | China | B1 | / |
| *H. vulgaris* | Xu4595 | 50.20 | 119.48 | China | B1 | / |
| *H. vulgaris* | Xu4601 | 49.16 | 120.33 | China | B1 | / |
| *H. vulgaris* | Xu4615 | 48.51 | 123.82 | China | B1 | / |
| *H. vulgaris* | Xu4618 | 47.53 | 122.20 | China | B1 | / |
| *H. vulgaris* | Xu5022 | 37.25 | 97.03 | China | B1 | / |
| *H. vulgaris* | Xu5106 | 30.00 | 89.10 | China | B1 | / |
| *H. vulgaris* | Xu5127 | 29.42 | 85.24 | China | B1 | / |
| *H. vulgaris* | Xu6207 | 47.41 | 119.62 | China | B1 | / |
| *H. vulgaris* | Xu6568 | 35.62 | 106.10 | China | B1 | / |
| *H. vulgaris* | Xu6613 | 38.30 | 102.01 | China | B1 | / |
| *H. vulgaris* | Xu6624 | 38.92 | 100.61 | China | B1 | / |
| *H. vulgaris* | Xu6669 | 40.25 | 99.43 | China | B1 | / |
| *H. vulgaris* | Xu6751 | 40.64 | 110.94 | China | B1 | / |
| *H. vulgaris* | Xu6776 | 45.84 | 119.32 | China | B1 | / |
| *H. vulgaris* | Xu6786 | 44.21 | 120.37 | China | B1 | / |
| *H. vulgaris* | Xu7192 | 49.49 | 121.36 | China | B1 | / |
| *H. vulgaris* | Xu7196 | 49.28 | 119.29 | China | B1 | / |
| *H. vulgaris* | Xu7219 | 47.67 | 119.28 | China | B1 | / |
| *H. vulgaris* | Xu462 | 49.58 | 124.72 | China | B2 (OK500324) | II |
| *H. vulgaris* | Xu4036 | 39.18 | 100.14 | China | B2 | / |
| *H. vulgaris* | Xu5197 | 38.87 | 112.21 | China | B2 | / |
| *H. vulgaris* | Xu2349 | 30.16 | 101.50 | China | B3 (OK500325) | II |
| *H. vulgaris* | Xu2493 | 30.48 | 91.10 | China | B3 | II |
| *H. vulgaris* | Xu5124 | 28.59 | 86.83 | China | B3 | II |
| *H. vulgaris* | Xu5142 | 33.17 | 79.84 | China | B3 | II |
| *H. vulgaris* | Xu2470 | 29.63 | 94.38 | China | B3 | / |
| *H. vulgaris* | Xu2504 | 33.49 | 100.05 | China | B3 | / |
| *H. vulgaris* | Xu5103 | 30.75 | 88.79 | China | B4 (OK500326) | / |
| *H. vulgaris* | Xu7200 | 49.49 | 117.76 | China | B5 (OK500327) | / |
| *H. vulgaris* | V. Novikov 5881 | 54.33 | 40.63 | Russia | A1 | III |
| *H. vulgaris* | A. Bobrov s.n. | 69.24 | 33.23 | Russia | A1 | I |
| *H. vulgaris* | P. Volkova s.n. | 68.50 | 112.49 | Russia | A1 | I |
| *H. vulgaris* | S. Rosbakh s.n. | 50.91 | 107.08 | Russia | A1 | / |
| *H. vulgaris* | A.S. Baikalova s.n. | 60.50 | 74.05 | Russia | A1 | I |
| *H. vulgaris* | L. Abramova s.n. | 67.15 | 32.91 | Russia | A1 | I |
| *H. vulgaris* | L. Abramova s.n. | 66.41 | 33.69 | Russia | A1 | / |
| *H. vulgaris* | A.P. Belavskaya s.n. | 59.56 | 30.11 | Russia | A1 | / |
| *H. vulgaris* | L. Abramova s.n. | 64.35 | 40.71 | Russia | A1 | / |
| *H. vulgaris* | V. Ekzertsev s.n. | 57.88 | 55.36 | Russia | A1 | / |
| *H. vulgaris* | V. Papchenkov s.n. | 57.54 | 38.04 | Russia | A1 | I+II |
| *H. vulgaris* | L. Abramova s.n. | 57.81 | 35.03 | Russia | A1 | I |
| *H. vulgaris* | M. Krasnova s.n. | 66.69 | 38.88 | Russia | A4 (OK500320) | I |
| *H. vulgaris* | A.N. Efremov s.n. | 62.53 | 113.94 | Russia | A5 (OK500321) | I |
| *H. vulgaris* | N. Vodopyanova 210 | 55.97 | 113.85 | Russia | A6 (OK500322) | / |
| *H. vulgaris* | V. Chepinoga s.n. | 50.34 | 115.40 | Russia | B1 | / |
| *H. vulgaris* | V. Papchenkov s.n. | 52.86 | 53.02 | Russia | B1 | I+II |
| *H. vulgaris* | A. Bobrov s.n. | 59.86 | 42.24 | Russia | B1 | / |
| *H. vulgaris* | N. Reshetnikova s.n. | 54.92 | 34.98 | Russia | B1 | / |
| *H. vulgaris* | V.N. Siplivinsky s.n. | 57.82 | 84.17 | Russia | B3 | / |
| *H. vulgaris* | M.A. Pureskin s.n. | 54.37 | 51.88 | Russia | B3 | / |
| *H. vulgaris* | 95-39332 | 52.63 | 104.71 | Russia | B6 (OK500328) | / |
| *H. vulgaris* | V.V. Petrovsky s.n. | 69.08 | 160.97 | Russia | C1 (OK500329) | / |
| *H. vulgaris* | T. Plieva s.n. | 67.36 | 168.33 | Russia | C1 | / |
| *H. vulgaris* | M. Mikhailova s.n. | 69.70 | 157.40 | Russia | C1 | / |
| *H. vulgaris* | R.E. Beschel 16634 | 62.03 | 129.76 | Russia | C1 | / |
| *H. vulgaris* | M. Mikhailova s.n. | 69.70 | 157.40 | Russia | C1 | / |
| *H. vulgaris* | M. Vasiloeva s.n. | 64.86 | 173.34 | Russia | C1 | / |
| *H. vulgaris* | O.A. Mochalova s.n. | 59.22 | 154.59 | Russia | C1 | I |
| *H. vulgaris* | O.A. Mochalova s.n. | 62.70 | 156.32 | Russia | C1 | I |
| *H. vulgaris* | O.A. Mochalova s.n. | 62.59 | 165.85 | Russia | C1 | I |
| *H. vulgaris* | R. Zaripov s.n. | 45.91 | 133.92 | Russia | C1 | / |
| *H. vulgaris* | R. Zaripov s.n. | 49.90 | 142.77 | Russia | C1 | I |
| *H. vulgaris* | N. Panarina s.n. | 66.56 | 32.29 | Russia | C1 | / |
| *H. vulgaris* | O.A. Mochalova s.n. | 62.43 | 156.55 | Russia | C3 (OK500331) | I |
| *H. vulgaris* | K.V. Plyavgo s.n. | 52.07 | 27.76 | Belarus | B1 | / |
| *H. vulgaris* | V. Papchenkov s.n. | 47.31 | 110.65 | Mongolia | B1 | / |
| *H. vulgaris* | V. Papchenkov s.n. | 47.76 | 102.74 | Mongolia | B1 | / |
| *H. vulgaris* | V. Papchenkov s.n. | 50.49 | 100.21 | Mongolia | B3 | / |
| *H. vulgaris* | B. Federley s.n. | 69.04 | 20.80 | Finland | A1 | / |
| *H. vulgaris* | R. Fagerstén s.n. | 62.76 | 27.69 | Finland | C1 | III |
| *H. vulgaris* | E. Kemppainen 386 | 60.43 | 26.41 | Finland | C1 | III |
| *H. vulgaris* | I. Kukkonen 9100 | 60.30 | 22.40 | Finland | C1 | / |
| *H. vulgaris* | A. Charpin 108 | 46.63 | 6.01 | France | A1 | III |
| *H. vulgaris* | Freeland E13 | 50.06 | 14.42 | Czech | B1 | II |
| *H. vulgaris* | Freeland E10 | 48.19 | 16.48 | Austria | B1 | II |
| *H. vulgaris* | Freeland E05 | 52.31 | 0.29 | UK | B1 | II |
| *H. vulgaris* | Freeland E02 | 52.19 | 0.12 | UK | B1 | II |
| *H. vulgaris* | Áskell 0304 | 63.95 | -21.00 | Iceland | A1 | III |
| *H. vulgaris* | Oldham 25002 | 55.03 | -85.43 | Canada | C2 (OK500330) | / |
| *H. vulgaris* | Oldham 41232 | 55.58 | -85.70 | Canada | C2 | III |
| *H. vulgaris* | Oldham 41382b | 55.24 | -84.32 | Canada | C2 | III |
| *H. vulgaris* | XUNA016 | 53.56 | -114.09 | Canada | C2 | / |
| *H. vulgaris* | XUNA059 | 50.42 | -96.58 | Canada | C2 | III |
| *H. vulgaris* | Bennett 17-0073 | 60.87 | -136.43 | Canada | C2 | / |
| *H. vulgaris* | Oldham 24621 | 55.23 | -85.13 | Canada | C1 | / |
| *H. vulgaris* | EAS0172 | 52.36 | -88.12 | Canada | C1 | III |
| *H. vulgaris* | XUNA048 | 48.62 | -90.52 | Canada | C1 | III |
| *H. vulgaris* | XUNA063 | 49.74 | -95.16 | Canada | C1 | III |
| *H. vulgaris* | Bennett 17-0481 | 63.89 | -135.71 | Canada | C1 | / |
| *H. vulgaris* | Consaul 4279 | 55.84 | -79.87 | Canada | C1 | / |
| *H. vulgaris* | Consaul 4326 | 55.91 | -79.61 | Canada | C1 | III |
| *H. vulgaris* | Gillespie 11319 | 61.10 | -94.02 | Canada | C1 | III |
| *H. vulgaris* | Saarela 3616 | 67.52 | -115.61 | Canada | C1 | / |
| *H. vulgaris* | J.K. Buttese 802 | 47.99 | -90.01 | USA | C1 | III |
| *H. vulgaris* | S. Goodrich 22981 | 40.90 | -109.94 | USA | C1 | III |
| *H. tetraphylla* | Oldham 40024 | 52.06 | -81.08 | Canada | C1 | III |
| *H. tetraphylla* | Oldham 40859a | 55.27 | -84.33 | Canada | C1 | III |
| *H. tetraphylla* | Bennett 15-0403 | 68.86 | -136.68 | Canada | C1 | III |
| *H. tetraphylla* | T. Ulvinen s.n. | 64.91 | 25.38 | Finland | C1 | / |
| *H. tetraphylla* | I. Kause s.n. | 61.40 | 21.53 | Finland | C1 | III |
| *H. tetraphylla* | T.M. Koroleva s.n. | 69.56 | 165.21 | Russia | C1 | / |
| *H. tetraphylla* | L. Sergienka 6334 | 62.96 | 179.07 | Russia | C1 | / |
| *H. tetraphylla* | N. Panarina s.n. | 66.68 | 32.87 | Russia | C1 | I |
| *H. tetraphylla* | L. Abramova s.n. | 66.41 | 33.72 | Russia | C1 | I |
| *H. tetraphylla* | O.A. Mochalova s.n. | 59.68 | 151.33 | Russia | C1 | I |
| *H. lanceolata* | R. Elven s.n. | 70.29 | 25.50 | Norway | A1 | III |
| *H. lanceolata* | Oldham 40799 | 55.24 | -84.32 | Canada | C2 | III |
| *H. lanceolata* | Oldham 40903 | 55.23 | -84.31 | Canada | C2 | / |
| *H. lanceolata* | Oldham 41261 | 55.92 | -86.94 | Canada | C1 | III |
| *H. lanceolata* | Bennett 13-0193 | 69.16 | -104.90 | Canada | C1 | III |
| *H. lanceolata* | Oldham 40859b | 55.27 | -84.33 | Canada | C1 | III |
| *H. lanceolata* | EAS0910 | 56.76 | -88.64 | Canada | C1 | III |
| *H. lanceolata* | Bennett 05-1020 | 68.93 | -137.21 | Canada | C1 | III |
| *H. lanceolata* | Bennett 05-0986 | 68.98 | -137.43 | Canada | C1 | III |
| *H. lanceolata* | Bennett 15-0372 | 69.08 | -137.89 | Canada | C1 | III |
| *H. lanceolata* | Bennett 15-0266 | 68.78 | -137.50 | Canada | C1 | / |
| *H. lanceolata* | Bennett 14-0462 | 68.00 | -103.70 | Canada | C1 | / |
| *H. lanceolata* | Gillespie 11011 | 61.10 | -94.00 | Canada | C1 | III |
| *H. lanceolata* | Gillespie 8420 | 69.12 | -105.04 | Canada | C1 | / |
| *H. lanceolata* | Gillespie 9406 | 69.60 | -123.15 | Canada | C1 | / |
| *H. lanceolata* | Saarela 2444 | 62.99 | -69.71 | Canada | C1 | III |
| *H. lanceolata* | Saarela 5110 | 63.71 | -68.37 | Canada | C1 | / |
| *H. lanceolata* | Sokoloff 859_1 | 81.83 | -71.47 | Canada | C1 | / |
| *H. lanceolata* | T. Kelso 131 | 65.72 | -167.98 | Alaska | C1 | III |
| *H. lanceolata* | K.L. Chambers 114 | 69.75 | -163.06 | Alaska | C1 | III |
| *H. lanceolata* | F.H. Bormann 69BC | 69.36 | -152.12 | Alaska | C1 | III |
| *H. lanceolata* | J.H. Thomas 6284 | 59.45 | -146.32 | Alaska | C1 | III |
| *H. lanceolata* | P. Gravesen 66-2529 | 59.90 | -43.72 | Greenland | C1 | III |
| *H. lanceolata* | C. Hansen 67-1956 | 59.90 | -44.35 | Greenland | C1 | III |
| *H. lanceolata* | C. Hansen 67-397 | 60.13 | -44.75 | Greenland | C1 | III |
| *H. lanceolata* | J. Johansen 452 | 61.63 | -49.00 | Greenland | C1 | III |
| *H. lanceolata* | M. Møller 424b | 68.50 | -51.87 | Greenland | C1 | III |
| *H. lanceolata* | H.M. Raup 509 | 72.23 | -23.92 | Greenland | C1 | III |
| *H. lanceolata* | K. Hansen 1966 | 60.93 | -46.80 | Greenland | C1 | III |
| *H. lanceolata* | C.A. Jørgensen s.n. | 60.50 | -45.33 | Greenland | C1 | III |
| *H. lanceolata* | G. Halliday 42/80 | 75.47 | -20.04 | Greenland | C1 | III |
| *H. lanceolata* | O.A. Mochalova s.n. | 59.57 | 151.33 | Russia | C1 | I |
| *H. lanceolata* | O.A. Mochalova s.n. | 59.57 | 151.28 | Russia | C1 | I |

**Supplementary Table 2.** The settings for all prior parameters used in DIY-ABC Random Forest analyses for demographic history of *Hippuris vulgaris*. N1-N5 is the effective population size; NA is the ancestral effective population size; N1b-N5b is the effective number of founders; t1-t5 is the time of introduction events translated into numbers of generations; db is the duration of the initial bottleneck in generations.

| **Demographical parameters** | **Marker parameters** |
| --- | --- |
| N1, uniform [1e2 – 1e4];  N2, uniform [1e2 – 1e4];  N3, uniform [1e2 – 1e4];  N4, uniform [1e2 – 1e4];  N5, uniform [1e2 – 1e4];  NA, uniform [1e2 – 1e4];  N1b, uniform [1e2 – 1e4];  N2b, uniform [1e2 – 1e4];  N3b, uniform [1e2 – 1e4];  N4b, uniform [1e2 – 1e4];  N5b, uniform [1e2 – 1e4];  t1, uniform [10 – 1e3];  t2, uniform [10 – 1e3];  t3, uniform [10 – 1e3];  t4, uniform [10 – 1e3];  t5, uniform [10 – 1e3];  db, uniform [10 – 1e3];  r, uniform [0.01 – 0.99];  t5>t4>t3>t2>t1>db | mean *μ*, uniform [10^-4^ – 10^-3^];  gam *μ*, gamma [10^-5^ – 10^-2^];  mean *P*, uniform [0.1 – 0.3];  gam *P*, gamma [0.01 – 0.90] |

**Supplementary Table 3.**  Linkage disequilibrium analysis among seven microsatellite loci measured by the values of r^2^. r^2^ = 0, no linkage disequilibrium; r^2^ = 1, complete linkage disequilibrium.

|  | Hpv75 | Hpv22 | Hpv37 | Hpv67 | Hpv11 | Hpv30 |
| --- | --- | --- | --- | --- | --- | --- |
| Hpv27 | 0.073 | 0.194 | 0.061 | 0.090 | 0.107 | 0.183 |
| Hpv75 | - | 0.101 | 0.026 | 0.071 | 0.045 | 0.095 |
| Hpv22 | - | - | 0.065 | 0.129 | 0.098 | 0.231 |
| Hpv37 | - | - | - | 0.039 | 0.034 | 0.043 |
| Hpv67 | - | - | - | - | 0.067 | 0.111 |
| Hpv11 | - | - | - | - | - | 0.111 |

**Supplementary Table 4.** The data of seven microsatellite loci obtained from 105 *Hippuris* samples

| Sample | Latitude | Longitude | Country/  Region | Microsatellite loci | | | | | | | | | | | | | | | | | | | | |
| --- | --- | --- | --- | --- | --- | --- | --- | --- | --- | --- | --- | --- | --- | --- | --- | --- | --- | --- | --- | --- | --- | --- | --- | --- |
|  |  |  |  | Hpv27 | | | Hpv75 | | | Hpv22 | | | Hpv37 | | | Hpv67 | | | Hpv11 | | | Hpv30 | | |
| Xu001 | 55.24 | -84.32 | Canada | 196 | 196 | | 260 | 269 | | 272 | 272 | | 378 | 397 | | 275 | 277 | | 332 | 332 | | 348 | 350 | |
| Xu003 | 55.92 | -86.94 | Canada | 196 | 196 | | 260 | 269 | | 272 | 272 | | - | - | | 277 | 277 | | 332 | 332 | | 348 | 350 | |
| Xu004 | 69.16 | -104.90 | Canada | 196 | 196 | | 257 | 257 | | 272 | 272 | | 378 | 397 | | 284 | 284 | | 334 | 334 | | 350 | 352 | |
| Xu005 | 55.27 | -84.33 | Canada | 196 | 196 | | 260 | 266 | | 272 | 272 | | 378 | 394 | | 277 | 277 | | 332 | 332 | | 348 | 350 | |
| Xu006 | 56.76 | -88.64 | Canada | 196 | 196 | | 260 | 260 | | 272 | 272 | | 367 | 396 | | 275 | 284 | | 332 | 332 | | 348 | 350 | |
| Xu007 | 52.06 | -81.08 | Canada | 196 | 196 | | 260 | 263 | | 272 | 272 | | 367 | 394 | | 284 | 284 | | 320 | 332 | | 350 | 350 | |
| Xu008 | 55.27 | -84.33 | Canada | 196 | 196 | | 260 | 260 | | 272 | 272 | | 367 | 394 | | 277 | 277 | | 320 | 332 | | 350 | 350 | |
| Xu014 | 55.58 | -85.70 | Canada | 196 | 196 | | 260 | 266 | | 272 | 272 | | 367 | 378 | | 275 | 277 | | 332 | 332 | | 346 | 346 | |
| Xu016 | 55.24 | -84.32 | Canada | 196 | 196 | | 266 | 269 | | 272 | 272 | | 378 | 401 | | 275 | 277 | | 316 | 332 | | 348 | 348 | |
| Xu018 | 52.36 | -88.12 | Canada | 196 | 196 | | 263 | 263 | | 272 | 272 | | 378 | 397 | | 284 | 284 | | 334 | 334 | | 350 | 350 | |
| Xu041 | 68.86 | -136.68 | Canada | 196 | 196 | | 260 | 260 | | 272 | 272 | | 367 | 394 | | 284 | 284 | | 332 | 332 | | 350 | 350 | |
| Xu042 | 68.93 | -137.21 | Canada | 177 | 196 | | 257 | 260 | | 272 | 272 | | 378 | 394 | | 280 | 284 | | 332 | 332 | | 350 | 350 | |
| Xu043 | 68.98 | -137.43 | Canada | 196 | 196 | | 260 | 260 | | 272 | 272 | | 378 | 378 | | 284 | 284 | | 316 | 332 | | 350 | 352 | |
| Xu044 | 69.08 | -137.89 | Canada | 177 | 196 | | 257 | 260 | | 272 | 272 | | 391 | 391 | | 289 | 289 | | 316 | 332 | | 350 | 350 | |
| Xu055 | 48.62 | -90.52 | Canada | 196 | 196 | | 263 | 263 | | 272 | 272 | | 382 | 400 | | 284 | 284 | | 316 | 332 | | 350 | 350 | |
| Xu056 | 50.42 | -96.58 | Canada | 196 | 196 | | 260 | 260 | | 272 | 272 | | 378 | 400 | | 266 | 275 | | 332 | 332 | | - | - | |
| Xu057 | 49.74 | -95.16 | Canada | 196 | 196 | | 257 | 263 | | 272 | 272 | | 378 | 400 | | 277 | 284 | | 334 | 334 | | - | - | |
| Xu106 | 55.91 | -79.61 | Canada | 196 | 196 | | 257 | 266 | | 265 | 272 | | 368 | 402 | | 275 | 277 | | 316 | 334 | | 348 | 350 | |
| Xu107 | 61.10 | -94.00 | Canada | 196 | 196 | | 257 | 260 | | 272 | 272 | | 378 | 397 | | 284 | 284 | | 334 | 334 | | 350 | 352 | |
| Xu108 | 61.10 | -94.02 | Canada | 168 | 196 | | 236 | 257 | | 265 | 272 | | 368 | 380 | | 277 | 284 | | 316 | 334 | | 350 | 350 | |
| Xu111 | 62.99 | -69.71 | Canada | 196 | 196 | | 257 | 257 | | 265 | 272 | | 368 | 380 | | 277 | 284 | | 334 | 334 | | 350 | 350 | |
| Xu135 | 65.72 | -167.98 | Alaska | 177 | 177 | | 257 | 257 | | 272 | 272 | | 367 | 379 | | 277 | 284 | | 334 | 334 | | 350 | 350 | |
| Xu136 | 69.75 | -163.06 | Alaska | 177 | | 196 | 257 | | 257 | 272 | | 272 | 379 | | 379 | 284 | | 284 | 332 | | 334 | - | | - |
| Xu137 | 69.36 | -152.12 | Alaska | 177 | | 196 | 257 | | 257 | 272 | | 272 | 379 | | 414 | 284 | | 284 | 334 | | 334 | 350 | | 350 |
| Xu138 | 59.45 | -146.32 | Alaska | 196 | | 211 | 257 | | 263 | 272 | | 286 | 366 | | 378 | 277 | | 277 | - | | - | 355 | | 355 |
| Xu133 | 40.90 | -109.94 | USA | - | | - | 265 | | 265 | 272 | | 286 | 378 | | 401 | 275 | | 284 | 314 | | 331 | 355 | | 355 |
| Xu132 | 47.99 | -90.01 | USA | 196 | | 211 | 257 | | 257 | 272 | | 286 | 366 | | 378 | 284 | | 284 | 314 | | 331 | - | | - |
| Xu119 | 59.90 | -44.35 | Greenland | 196 | | 196 | 263 | | 263 | 272 | | 272 | 378 | | 403 | 283 | | 283 | 316 | | 334 | 350 | | 350 |
| Xu121 | 61.63 | -49.00 | Greenland | 177 | | 177 | 257 | | 257 | 272 | | 272 | 367 | | 378 | 277 | | 277 | 316 | | 334 | 352 | | 352 |
| Xu122 | 68.50 | -51.87 | Greenland | 196 | | 196 | 257 | | 257 | 272 | | 272 | 378 | | 378 | 284 | | 284 | 316 | | 334 | 352 | | 352 |
| Xu125 | 60.50 | -45.33 | Greenland | 196 | | 196 | 257 | | 257 | 272 | | 272 | 379 | | 379 | 277 | | 277 | 316 | | 334 | 352 | | 352 |
| Xu126 | 75.47 | -20.04 | Greenland | 196 | | 196 | 257 | | 263 | 272 | | 272 | 378 | | 378 | 277 | | 277 | 334 | | 334 | 348 | | 352 |
| Xu118 | 59.90 | -43.72 | Greenland | 196 | | 196 | 263 | | 263 | 272 | | 272 | 378 | | 397 | 284 | | 284 | 334 | | 334 | 350 | | 350 |
| Xu120 | 60.13 | -44.75 | Greenland | 196 | | 196 | 257 | | 257 | 272 | | 286 | 366 | | 378 | 277 | | 284 | 314 | | 333 | - | | - |
| Xu123 | 72.23 | -23.92 | Greenland | 196 | | 196 | 257 | | 257 | 272 | | 286 | 366 | | 378 | 277 | | 284 | 314 | | 333 | 355 | | 355 |
| Xu124 | 60.93 | -46.80 | Greenland | 177 | | 177 | 257 | | 265 | 272 | | 286 | 366 | | 378 | 277 | | 277 | 314 | | 333 | 355 | | 355 |
| Xu140 | 62.76 | 27.69 | Finland | 196 | | 196 | 260 | | 260 | 272 | | 272 | 379 | | 395 | 284 | | 284 | 334 | | 334 | 346 | | 346 |
| Xu143 | 60.43 | 26.41 | Finland | 196 | | 196 | 260 | | 260 | 286 | | 286 | 366 | | 393 | 284 | | 284 | - | | - | 355 | | 355 |
| Xu131 | 61.40 | 21.53 | Finland | 196 | | 211 | 260 | | 265 | 272 | | 286 | 366 | | 378 | 284 | | 284 | 314 | | 331 | 355 | | 355 |
| Xu127 | 70.29 | 25.50 | Norway | 196 | | 196 | 260 | | 263 | 272 | | 272 | 367 | | 367 | 284 | | 284 | 332 | | 332 | 350 | | 350 |
| Xu147 | 63.95 | -21.00 | Iceland | 196 | | 196 | 260 | | 260 | 272 | | 272 | 379 | | 397 | 284 | | 284 | 334 | | 334 | - | | - |
| Xu154 | 46.63 | 6.01 | France | 196 | | 211 | 260 | | 263 | 272 | | 272 | 395 | | 395 | 284 | | 284 | - | | - | 350 | | 350 |
| Xu047 | 48.19 | 16.48 | Austria | 211 | | 211 | 266 | | 266 | 286 | | 286 | 382 | | 396 | 294 | | 294 | 338 | | 338 | 355 | | 355 |
| Xu049 | 52.31 | 0.29 | UK | 211 | | 211 | 266 | | 285 | 286 | | 294 | 382 | | 396 | 286 | | 294 | 338 | | 338 | 355 | | 355 |
| Xu050 | 52.19 | 0.12 | UK | 211 | | 211 | 266 | | 285 | 286 | | 294 | 384 | | 396 | 286 | | 294 | 338 | | 338 | 355 | | 355 |
| Xu048 | 50.06 | 14.42 | Czech | 211 | | 211 | 266 | | 266 | 286 | | 292 | 382 | | 394 | 302 | | 302 | 338 | | 338 | 355 | | 355 |
| Hip21 | 52.86 | 53.02 | Russia | 211 | | 211 | 266 | | 266 | 272 | | 284 | 394 | | 394 | 302 | | 302 | 315 | | 333 | 355 | | 355 |
| Xu155 | 54.33 | 40.63 | Russia | 196 | | 211 | 257 | | 260 | 272 | | 272 | 397 | | 397 | 284 | | 294 | 334 | | 334 | 350 | | 350 |
| Hip36 | 57.54 | 38.04 | Russia | 211 | | 211 | 260 | | 266 | 272 | | 272 | 378 | | 396 | 284 | | 302 | 315 | | 333 | 349 | | 355 |
| Hip08 | 69.24 | 33.23 | Russia | 211 | | 211 | 260 | | 263 | 272 | | 284 | 366 | | 396 | 284 | | 294 | 315 | | 315 | 349 | | 349 |
| Hip07 | 66.69 | 38.88 | Russia | 211 | | 211 | 257 | | 257 | 272 | | 284 | 378 | | 396 | 284 | | 294 | 333 | | 333 | 349 | | 349 |
| Hip27 | 67.15 | 32.91 | Russia | 211 | | 211 | 263 | | 263 | - | | - | 366 | | 378 | 284 | | 284 | 315 | | 333 | 349 | | 349 |
| Hip37 | 57.81 | 35.03 | Russia | 211 | | 211 | 260 | | 260 | - | | - | 378 | | 396 | 284 | | 284 | 315 | | 315 | 349 | | 349 |
| Hip01 | 66.68 | 32.87 | Russia | 195 | | 211 | 260 | | 260 | 272 | | 272 | 366 | | 394 | 284 | | 284 | - | | - | 349 | | 349 |
| Hip02 | 66.41 | 33.72 | Russia | 195 | | 211 | 260 | | 260 | 272 | | 272 | 366 | | 394 | 284 | | 284 | - | | - | 349 | | 349 |
| Hip05 | 59.57 | 151.33 | Russia | 195 | | 211 | 263 | | 266 | 272 | | 272 | 366 | | 394 | 278 | | 278 | 330 | | 330 | 349 | | 349 |
| Hip09 | 59.22 | 154.59 | Russia | 211 | | 211 | 257 | | 257 | 272 | | 284 | 366 | | 396 | 284 | | 294 | 315 | | 333 | 349 | | 349 |
| Hip11 | 62.59 | 165.85 | Russia | 211 | | 211 | 257 | | 260 | 272 | | 272 | 378 | | 396 | 284 | | 294 | 315 | | 333 | 349 | | 355 |
| Hip03 | 59.68 | 151.33 | Russia | 195 | | 211 | 263 | | 263 | 272 | | 272 | 366 | | 394 | 278 | | 278 | 330 | | 330 | 349 | | 349 |
| Hip06 | 59.57 | 151.28 | Russia | 195 | | 211 | 263 | | 263 | 272 | | 272 | 366 | | 394 | 278 | | 278 | 330 | | 330 | 349 | | 349 |
| Hip13 | 49.90 | 142.77 | Russia | 211 | | 211 | 257 | | 257 | 272 | | 272 | 378 | | 396 | - | | - | 315 | | 315 | 349 | | 349 |
| Hip22 | 62.43 | 156.55 | Russia | 211 | | 211 | 257 | | 257 | 272 | | 272 | 378 | | 396 | 284 | | 284 | 315 | | 333 | 349 | | 349 |
| Hip10 | 62.70 | 156.32 | Russia | 195 | | 211 | 257 | | 257 | - | | - | 366 | | 394 | 284 | | 294 | 315 | | 333 | 349 | | 349 |
| Hip14 | 68.50 | 112.49 | Russia | 211 | | 211 | 260 | | 260 | - | | - | 378 | | 396 | 284 | | 294 | 315 | | 315 | 349 | | 349 |
| Hip20 | 60.50 | 74.05 | Russia | 211 | | 211 | 263 | | 263 | - | | - | 366 | | 378 | 284 | | 294 | 315 | | 333 | 349 | | 349 |
| Hip23 | 62.53 | 113.94 | Russia | 211 | | 211 | 260 | | 263 | 272 | | 272 | 378 | | 394 | 284 | | 284 | 315 | | 333 | 349 | | 349 |
| GH5 | 50.57 | 121.05 | China | 195 | | 211 | 260 | | 260 | 272 | | 272 | 380 | | 380 | 284 | | 284 | 333 | | 333 | 349 | | 355 |
| EEGN1 | 51.01 | 120.05 | China | 195 | | 195 | 260 | | 263 | 272 | | 272 | 379 | | 380 | 284 | | 284 | 333 | | 333 | 349 | | 349 |
| ELC1 | 50.67 | 122.13 | China | 195 | | 195 | 260 | | 260 | 272 | | 272 | 379 | | 379 | 284 | | 284 | 333 | | 333 | 349 | | 349 |
| GH4 | 50.77 | 121.50 | China | 195 | | 195 | 260 | | 260 | 272 | | 272 | 379 | | 380 | 284 | | 284 | 333 | | 333 | 349 | | 349 |
| YiC2 | 48.47 | 129.67 | China | 195 | | 195 | 257 | | 260 | 272 | | 272 | 379 | | 379 | 284 | | 284 | 333 | | 333 | 349 | | 349 |
| MH1 | 52.86 | 123.33 | China | 195 | | 195 | 260 | | 260 | 272 | | 272 | 379 | | 379 | 284 | | 288 | 333 | | 333 | 349 | | 349 |
| MH2 | 52.94 | 122.57 | China | 189 | | 195 | 260 | | 260 | 272 | | 272 | 379 | | 380 | 284 | | 288 | 333 | | 333 | 349 | | 349 |
| GH3 | 51.14 | 121.27 | China | 195 | | 195 | 260 | | 260 | 272 | | 272 | 379 | | 379 | 284 | | 284 | 333 | | 333 | 349 | | 349 |
| YKS2 | 50.15 | 121.83 | China | 195 | | 195 | 257 | | 260 | 272 | | 272 | 380 | | 380 | 284 | | 284 | 333 | | 333 | 349 | | 349 |
| HM2 | 51.84 | 124.54 | China | 195 | | 195 | 260 | | 260 | 272 | | 272 | 379 | | 380 | 284 | | 284 | 333 | | 333 | 349 | | 349 |
| AES2 | 46.71 | 120.72 | China | 195 | | 211 | 260 | | 272 | 272 | | 276 | 379 | | 379 | 284 | | 304 | 333 | | 339 | 349 | | 355 |
| KYQQ | 46.51 | 121.38 | China | 195 | | 211 | 260 | | 272 | 272 | | 276 | 379 | | 379 | 284 | | 304 | 333 | | 339 | 349 | | 355 |
| HM1 | 51.99 | 126.25 | China | 195 | | 211 | 260 | | 266 | 272 | | 282 | 396 | | 396 | 284 | | 304 | 333 | | 341 | 349 | | 355 |
| DC | 29.28 | 100.08 | China | 211 | | 211 | 266 | | 266 | 278 | | 278 | 400 | | 400 | 300 | | 300 | 335 | | 337 | 355 | | 355 |
| HY | 32.50 | 102.37 | China | 211 | | 211 | 266 | | 266 | 278 | | 284 | 400 | | 400 | 300 | | 304 | 335 | | 337 | 355 | | 355 |
| SX | 31.73 | 94.49 | China | 211 | | 211 | 266 | | 266 | 278 | | 282 | 400 | | 402 | 300 | | 300 | 335 | | 335 | 355 | | 355 |
| CN | 28.06 | 91.95 | China | 211 | | 211 | 266 | | 266 | 286 | | 292 | 400 | | 400 | 300 | | 300 | 337 | | 339 | 355 | | 355 |
| KD | 30.16 | 101.50 | China | 211 | | 211 | 269 | | 269 | 278 | | 286 | 398 | | 398 | 300 | | 300 | 341 | | 341 | 355 | | 355 |
| DX | 30.48 | 91.10 | China | 211 | | 211 | 266 | | 266 | 282 | | 286 | 398 | | 398 | 300 | | 300 | 339 | | 339 | 355 | | 355 |
| ABG | 43.33 | 115.68 | China | 211 | | 211 | 257 | | 266 | 286 | | 288 | 400 | | 400 | 304 | | 304 | 337 | | 339 | 355 | | 355 |
| QH | 46.49 | 90.20 | China | 211 | | 211 | 266 | | 272 | - | | - | - | | - | 304 | | 304 | 337 | | 339 | 355 | | 355 |
| MLDW | 49.58 | 124.72 | China | 211 | | 211 | 266 | | 266 | 276 | | 284 | 396 | | 396 | 304 | | 304 | 339 | | 339 | 355 | | 355 |
| JZG | 33.26 | 103.76 | China | 211 | | 211 | 257 | | 266 | 276 | | 276 | 396 | | 396 | 304 | | 304 | 339 | | 339 | 355 | | 355 |
| BLK | 43.63 | 93.19 | China | 211 | | 211 | 266 | | 266 | 286 | | 292 | 396 | | 396 | 304 | | 306 | 335 | | 339 | 355 | | 355 |
| EM | 46.67 | 83.93 | China | 211 | | 211 | 269 | | 269 | 292 | | 292 | 398 | | 398 | 304 | | 304 | 339 | | 339 | 355 | | 355 |
| ZS | 43.16 | 81.50 | China | 211 | | 211 | 266 | | 266 | 296 | | 296 | 396 | | 396 | 296 | | 296 | 339 | | 339 | 355 | | 355 |
| SW | 44.51 | 86.00 | China | 211 | | 211 | 269 | | 269 | 288 | | 292 | 396 | | 398 | 296 | | 304 | 339 | | 339 | 355 | | 355 |
| MY | 37.61 | 101.32 | China | 211 | | 211 | 266 | | 266 | 288 | | 288 | 396 | | 396 | 304 | | 304 | 339 | | 339 | 355 | | 355 |
| HX | 37.54 | 95.43 | China | 211 | | 211 | 266 | | 266 | 288 | | 288 | 396 | | 396 | 304 | | 304 | 339 | | 339 | 355 | | 355 |
| MD | 34.72 | 98.10 | China | 211 | | 211 | 266 | | 266 | 276 | | 278 | 400 | | 400 | 304 | | 304 | 339 | | 339 | 355 | | 355 |
| DiR | 28.59 | 86.83 | China | 211 | | 211 | 266 | | 272 | 292 | | 292 | 396 | | 396 | 300 | | 304 | 339 | | 339 | 355 | | 355 |
| PL | 30.81 | 81.56 | China | 211 | | 211 | 266 | | 269 | 288 | | 292 | 398 | | 398 | 296 | | 296 | 339 | | 339 | 355 | | 355 |
| RT | 33.17 | 79.84 | China | 211 | | 211 | 266 | | 269 | 282 | | 288 | 396 | | 396 | 296 | | 304 | 339 | | 339 | 355 | | 355 |
| TZ | 37.38 | 102.96 | China | 211 | | 211 | 272 | | 272 | 280 | | 284 | 396 | | 396 | 304 | | 304 | 339 | | 339 | 355 | | 355 |
| JQ | 39.74 | 98.55 | China | 211 | | 211 | 266 | | 266 | 284 | | 284 | 396 | | 396 | 304 | | 304 | 339 | | 339 | 355 | | 355 |
| ZZ | 40.88 | 112.78 | China | 211 | | 211 | 266 | | 269 | 276 | | 276 | 396 | | 396 | 304 | | 304 | 339 | | 339 | 355 | | 355 |
| WNT | 43.23 | 119.55 | China | 211 | | 211 | 266 | | 272 | 276 | | 276 | 396 | | 396 | 304 | | 306 | 339 | | 341 | 355 | | 355 |
| CGL | 45.20 | 124.45 | China | 211 | | 211 | 266 | | 266 | 274 | | 288 | 396 | | 396 | 304 | | 304 | 339 | | 339 | 355 | | 355 |
| XYQ | 48.41 | 117.57 | China | 211 | | 211 | 272 | | 272 | 284 | | 288 | 396 | | 396 | 304 | | 304 | 339 | | 341 | 355 | | 355 |
